# Increases in temperature do not translate to increased flooding

**DOI:** 10.1038/s41467-019-13612-5

**Published:** 2019-12-12

**Authors:** Conrad Wasko, Ashish Sharma, Dennis P. Lettenmaier

**Affiliations:** 1 Department of Infrastructure Engineering, The University of Melbourne, Melbourne, VIC 3010 Australia; 2 Department of Environmental Sciences, University of California Riverside, Riverside, CA 92521 USA; 3 Civil and Environmental Engineering, The University of New South Wales, Sydney, NSW 2052 Australia; 4 Department of Geography, University of California Los Angeles, Los Angeles, CA 90095 USA

**Keywords:** Climate sciences, Environmental sciences, Hydrology, Natural hazards

**Arising from** Guo et al. *Nature Communications* 10.1038/s41467-018-06765-2

In a recently published study and subsequent correspondence Yin et al.^1,2^ examine the sensitivity of precipitation and streamflow with temperature for the 99th percentile of precipitation and streamflow. The sensitivity of streamflow with temperature is found to be greater than the Clausius-Clapeyron (CC) relation of ~7% °C^−1^ and greater than the precipitation-temperature sensitivity. As a result, they conclude that storm runoff, in particular flash flooding, may increase (at more than the CC rate) in response to climate change. Although the authors state that similar studies have not been performed, an almost identical global study published over a year ago found largely contradictory results^3^. The Yin et al^1^ results oppose the prevailing tide of literature that suggests global streamflow extremes are more likely to be decreasing than increasing with climatic change^4–7^. Here we argue that the Yin et al^1^ results do not support the assertion that flash flooding will increase with climatic change, but rather are more likely to be related to changes in snowmelt processes.

The analysis presented by Yin et al.^1^ is not hydrologically consistent with how flooding, and in particular, flash flooding occurs. Flash flooding is the result of short, intense bursts of rainfall over a relatively small area that are followed almost immediately (within minutes or hours) by a corresponding increase in discharge^8^. Nonetheless, the authors calculate sensitives using the entire streamflow record that is measured on a daily time step.

Furthermore, although a peak in a streamflow record may be physically related to temperature through the implied dependence of increased rainfall due to the Clausius-Clapeyron relationship, this physical dependence, for the 99th percentile of streamflow, will largely be modulated by the preceding soil moisture conditions^9^. Once the peak flow has been reached there will be a long recession curve, which is largely dependent on the catchment properties^10^ and is not a function of the temperature associated with the precipitation. For that reason, the Yin et al.^1^ analysis would better have been performed using flood peaks, and preferably peaks matched to rainfall peaks to ensure that the dependence between the precipitation, streamflow, soil moisture, and temperature is maintained^3^.

In presenting their results, Yin et al.^1^ presuppose that streamflow and precipitation both must increase together with increases in temperature. But these idealized relationships do not hold for much of the data presented. To ensure positive scaling, Yin et al.^1^ only perform analysis up to a maximum (peak point) temperature. However, Figure 2a, b Yin et al.^1^ present different peak point temperatures for precipitation and streamflow. As a result, the authors have used two different temperature ranges for their analysis, one for the sensitivity of precipitation to temperature, and another for the sensitivity of streamflow to temperature, with much colder temperatures used for the calculation of the streamflow sensitivity. We present previously published results^3^ to show how the use of different temperature ranges for the analysis of precipitation and streamflow bias their outcome.

Similar to the analysis presented in Yin et al.^1^ we matched streamflow peaks to local temperatures. Using temperatures above 5 °C, to remove the influence of snowmelt results in mostly negative streamflow sensitivities with temperature (Fig. 1). As the majority of the data presented in Yin et al.^1^ are from the U.S. and Europe, we focus on these two regions in Fig. 1b, 1c to show why different temperature ranges in the analysis of precipitation and streamflow can cause misleading results.

**Fig. 1:**
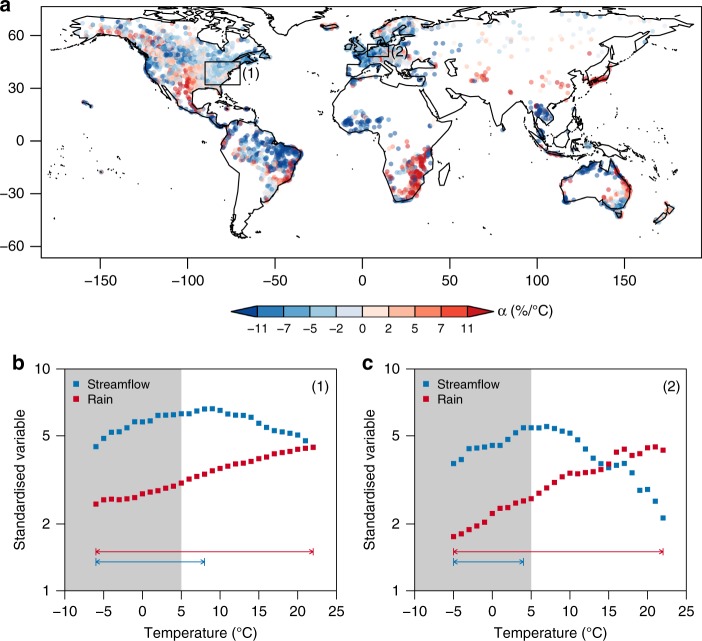
**Global streamflow and temperature scaling.** **a** Streamflow scaling with temperature for the 99th perceFig.above 5 °C. **b** Aggregation of data for North East America shown by region (1), binned using 2 °C temperature bins (**c**) similar results as in **b** but for central Europe shown by region (2). The horizontal lines show the temperature range that would be included when analysis is performed up to a peak point. The grey shading shows the temperature range likely to be affected by snow melt.

Using 2 °C temperature bins, the 99th percentile of precipitation monotonically increases with temperature (Fig. 1b, c), hence the temperature range analysed in Yin et al.^1^ corresponds to the entire record (red arrow). However, a peak point in the streamflow occurs at a relatively low temperature in both regions and hence the range of the temperature record (blue arrow) over which monotonic increases occurs is relatively small, and, is mostly likely to be snow-affected. Stated otherwise, whereas the majority of the temperature range for the precipitation analysis lies outside that influenced by snowmelt, the temperature range used for the streamflow analysis lies almost wholly inside the region influenced by snow processes^11^. This leads to very biased results, as the climatic processes captured by the precipitation scaling are vastly different to the processes involved in the streamflow scaling. Below the peak point temperature, increases in streamflow are likely due to the interaction of snow, rain, and warmer temperatures^12^, but above this, although rainfall continues to increase soil moisture begins to decrease reducing streamflow^3^.

Flash flooding will likely increase due to increased urbanisation and increased rainfalls^13,14^, and increases in streamflow above increases in rainfall are possible if, for example, the soil moisture conditions preceding streamflow events increase, but the results presented by Yin et al.^1^ do not support this assertion. With the use of temperature sensitivities for projection of climatic change remaining speculative^15^, the analysis Yin et al.^1^ present shows that, for most of the data they analyse, with higher temperatures there are increases in streamflow due to hydrologic processes involving snow^12^.

## Data Availability

The data used herein were used and referenced in published work by Wasko and Sharma^3^. Daily precipitation was obtained from the Global Historical Climatology Network [available at: 10.7289/V5D21VHZ], daily streamflow from the Global Runoff Data Centre [available at https://www.bafg.de/GRDC/], and daily gridded surface temperatures from Berkeley Earth [available at http://berkeleyearth.org/data/]
